# Occupational Therapy Interventions in Mental Health During Lockdown: A Scoping Review

**DOI:** 10.3390/healthcare13172136

**Published:** 2025-08-27

**Authors:** Laura-María Compañ-Gabucio, Gema Moreno-Morente, Verónica Company-Devesa, Laura Torres-Collado, Manuela García-de-la-Hera

**Affiliations:** 1 Departamento de Patología y Cirugía, Universidad Miguel Hernández (UMH), 03550 Alicante, Spain; lcompan@umh.es (L.-M.C.-G.);; 2 Instituto de Investigación Sanitaria y Biomédica de Alicante (ISABIAL), 03010 Alicante, Spain; 3 Unidad de Epidemiología de la Nutrición, Departamento de Salud Pública, Historia de la Ciencia y Ginecología, Universidad Miguel Hernández (UMH), 03550 Alicante, Spain; 4 CIBER Epidemiología y Salud Pública (CIBERESP), Instituto de Salud Carlos III, 28029 Madrid, Spain; 5 Being + Doing & Becoming Occupational Research Group (B+D+b), Miguel Hernández University, 03550 Alicante, Spain

**Keywords:** review, mood, daily life activities, telerehabilitation, occupation

## Abstract

Lockdown derived from the COVID-19 pandemic posed significant challenges to mental health care, prompting the adaptation of therapeutic practices. The objective of this study was to describe the characteristics and objectives of occupational therapy (OT) interventions conducted in the field of mental health during the COVID-19 lockdown. A scoping review was conducted following PRISMA-ScR guidelines. A systematic search was carried out in the following databases: PubMed, Scopus, Embase, OTSeeker, PsycINFO, and Web of Science. We included randomized or non-randomized intervention studies, published in English or Spanish, that explored OT interventions in mental health during the COVID-19 period and/or lockdown. Data were extracted using pre-designed tables in accordance with the recommendations of the Cochrane Handbook. We included seven articles. OT interventions were conducted via video call (n = 4) and in person (n = 3). These were carried out with adults, adolescents, and children, lasting from 1 to 32 weeks, with the number of sessions ranging from 7 to 22 and lasting 20 to 90 min. The most frequently addressed outcomes were quality of life (n = 4), anxiety or depression (n = 4), and sleep (n = 4). During lockdown, OT interventions were mainly applied via telerehabilitation with the aim of increasing activity participation and addressing emotional issues. These results could help occupational therapists to implement mental health interventions when in-person application is compromised.

## 1. Introduction

Coronavirus disease 2019 (COVID-19) is caused by severe acute respiratory syndrome [1,2,3,4]. The most common symptoms are fever, persistent cough, fatigue, shortness of breath, muscle pain, and headache, although some people are asymptomatic [5]. The effect of COVID-19 is more severe in individuals with chronic conditions such as hypertension, diabetes, or cancer, as well as older adults, and can even lead to death [6,7].

On 30 January 2020, the World Health Organization (WHO) announced a public health emergency of international concern related to COVID-19, which was elevated to a pandemic on 11 March 2020 [1]. To limit the spread of COVID-19, various preventive measures were implemented, such as the use of masks, hand sanitizers, social distancing, and lockdown [1,8]. This global lockdown had a significant impact on people’s lives, causing changes in education, work, well-being, quality of life, and health care [9,10]. In-person medical care was restricted, causing delays in diagnoses and treatments, and the conditions of certain individuals, especially those with mental disorders, worsened or were exacerbated [2,11].

Telerehabilitation emerged as an alternative to in-person therapies, serving as an intervention method to treat patients while aiming to reduce the risk of contagion and protect both patients and health care professionals [2,9,11]. In the case of occupational therapy (OT), a previous study has shown that occupational therapists implemented telerehabilitation and conducted consultations via videoconferencing or telephone [12]. It was observed that OT telerehabilitation provides the same treatment benefits as in-person therapies as well as preventing relapses in patients with severe mental disorders [12]. In this regard, Sánchez-Guarnido [12] showed that videoconferencing facilitated the therapeutic relationship more effectively than telephone calls for individuals with mental health issues, as face-to-face interaction strengthened the bond between the professional and the patient. Similarly, a recent study based on a global survey of occupational therapists showed that the main rehabilitation interventions provided by OT during the COVID-19 pandemic were related to cognition, fatigue management, self-regulation, relaxation, environmental adaptation, and mental health [13]. Thus, occupational therapists can use telerehabilitation to prevent the onset of mental health problems and support recovery [12,14]. However, these interventions face structural limitations such as technological barriers and reduced accessibility for vulnerable populations, which should be carefully considered when implementing telerehabilitation programs [15].

COVID-19 affects people’s health by causing deficits at functional, cardiac, psychological, neurological, and cognitive levels [9,10], impacting the performance of daily activities and mobility, which can lead to significant changes in daily life and behavior patterns [3,12]. However, there is little scientific evidence on the treatments provided by OT in the field of mental health issues because of COVID-19. Most reviews focused on the COVID-19 lockdown are aimed at describing its impact on the population’s mental health [16,17,18,19,20,21,22]. We found only one review that described interventions conducted during the lockdown, which focused on optimizing mental health during the COVID-19 pandemic but did not address OT interventions [23]. Therefore, our research question was: Which interventions were carried out in the field of mental health by occupational therapists during the lockdown according to the available scientific evidence? A scoping review was conducted to describe the characteristics and objectives of the interventions performed in the field of mental health by occupational therapists during the lockdown.

## 2. Materials and Methods

A scoping review was systematically conducted following the guidelines of the Cochrane Handbook [24] and the recommendations of the PRISMA extension for Scoping Reviews (PRISMA-ScR) [25] (Table S1). The review was carried out in five steps: literature search, article screening, study selection, data extraction, and synthesis of results. No protocol for this review was published, nor was it registered in PROSPERO or similar.

### 2.1. Search Strategy

On 8 March 2025, six databases were consulted following the recommendations of Bramer et al. [26]: PubMed, Embase, Scopus, PsycINFO, OTSeeker, and Web of Science. The same search terms were used across all the databases, combined with the Boolean operator AND, resulting in the following search strategy: “occupational therapy” AND “mental health” AND COVID. No time or study type filters were used in the literature search on any of the consulted databases. All search strategies are shown in Table 1.

### 2.2. Eligibility Criteria

The eligibility criteria were defined according to the PCC framework (Population, Concept, and Context) [27]:

Population/Participants: Individuals of all ages who received OT interventions during COVID-19 and/or its lockdown.

Concept: Interventions delivered by occupational therapists, either exclusively or in collaboration with other professionals, targeting mental health outcomes.

Context: Interventions conducted during COVID-19 and/or its lockdown.

Specifically, articles had to meet the following criteria in order to be included in this review:

- Studies published in English or Spanish.
- Studies with full text available.
- Studies conducted by occupational therapists, either exclusively or together with other professionals.
- Studies in which an intervention during COVID-19 and/or its lockdown was carried out.
- Studies where the intervention targeted mental health, including anxiety, depression, well-being, social stress, quality of life, or motivation.
- Studies with experimental or quasi-experimental designs.

Articles were excluded if they met the following criteria:

- Studies published in other languages.
- Studies whose intervention was not focused on mental health.
- Studies with a team which did not include an occupational therapist.
- Studies with an intervention period outside the COVID-19 timeframe.
- Studies with the following designs: abstracts, editorials, letters to the editor, opinions, reviews, brief reports, conference papers, books, book chapters, scale validation studies, qualitative studies, mixed methods studies, case reports, animal studies, pilot studies, case studies, observational studies, protocols, and exploratory studies.

All inclusion and exclusion criteria were applied manually.

### 2.3. Study Selection

The titles of articles obtained from the bibliographic searches in the six databases were downloaded into a Microsoft Excel spreadsheet. A preliminary selection was performed to remove duplicate articles. A two-step screening was then conducted: first, by title and abstract, and second, by full text. An exhaustive review and selection of articles were performed, discarding those that did not meet the inclusion criteria. One researcher (LMCG) created the Excel database and removed duplicates; two researchers (GMM and LMCG) independently screened the articles. In cases of disagreement, the inclusion criteria were jointly revisited and discussed. If consensus could not be reached, a third researcher (LTC) acted as arbitrator, applying the predefined eligibility criteria to reach a final decision.

### 2.4. Data Extraction and Synthesis

Before data extraction, tables were designed to collect information from the articles, and items to be included were defined in order to facilitate a more objective data synthesis and to avoid information manipulation. Based on the recommendations of the Cochrane Handbook version 6.4 [24], three tables were designed and completed. The first, “General Characteristics of Included Studies,” contained the PICOS parameters (Population, Intervention, Comparator, Outcome, and Study Design) for each study: author/year, study design, participants, intervention/comparison, study outcome and assessment tool. The second, “Summary of Findings,” provided information on characteristics directly related to our research question: author/year, intervention description, intervention duration, professionals involved in the intervention, and main results. The third, “Risk of Bias”, included author/year, main limitations, funding sources, and conflicts of interest of the included studies.

### 2.5. Quality Assessment

The quality of the included studies was not assessed as it is not a mandatory requirement in scoping reviews [28,29,30]. However, we included a table on the risk of bias which contains information on the main limitations, funding sources, and conflicts of interest reported by the included articles to allow readers to analyze the results of this work more critically. Additionally, the main limitations of the included articles are analyzed in the Section 3.

## 3. Results

**Figure 1:**
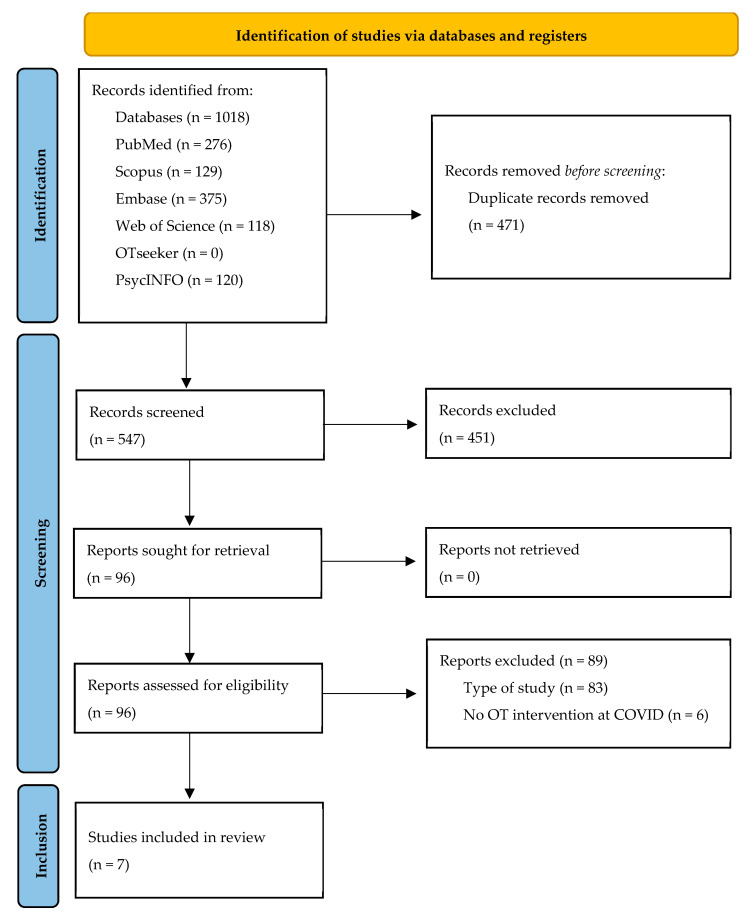
Flowchart of the study selection process.

A total of 1018 articles were obtained from the bibliographic searches conducted in the six databases consulted. Duplicates were removed, leaving 547 articles for screening by title and abstract. Of these, 96 articles were selected for full-text screening, and the 7 which met the inclusion criteria were included in this scoping review (Figure 1).

### 3.1. Main Characteristics of the Included Studies

**Figure 2:**
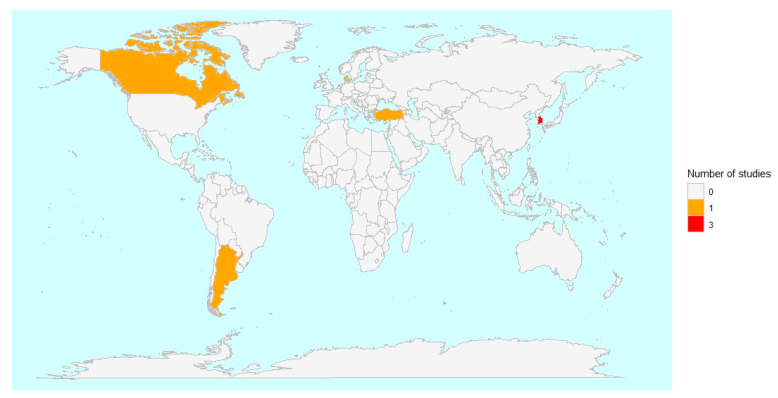
Geographical distribution of the studies included in this review.

The included articles were conducted in the following countries: South Korea (n = 3) [31,32,33], Canada (n = 1) [34], Turkey (n = 1) [35], Denmark (n = 1) [36], and Argentina (n = 1) [37] (Figure 2).

The studies were published in the years 2022 (n = 2) [31,35], 2023 (n = 2) [32,36], and 2024 (n = 4) [32,33,34,37]. The sample sizes ranged from 21 [34] to 139 participants [36]. Most articles included participants with an average age of ≥18 years (n = 5) [31,32,33,34,36], while a smaller number included participants with a younger average age (n = 2) [35,37]. The study populations comprised adults (n = 4) [31,32,33,36], children (n = 2) [35,37], and adolescents/young adults (n = 1) [34]. The interventions were carried out in refugee children [35] as well as in individuals with various pathologies such as physical disabilities [34], psychiatric disabilities [36], neurodevelopmental disorders and insomnia [37], or COVID-19 [31,32,33] (Table 2).

All the included articles were experimental studies with randomized (n = 5) [31,32,33,35,36] and non-randomized (n = 2) [34,37] designs. With the exception of one study [34], all the studies (n = 6) [31,32,33,35,36,37] included a control group (Table 2).

### 3.2. Occupational Therapy Interventions

Occupational therapy interventions carried out among the included studies are shown in Table 2. Most of the articles (n = 3) [31,35,36] compared the different interventions carried out in the intervention and control groups: OT via telerehabilitation + online classes versus only online classes [35], meaningful activities and recovery + standard mental health care versus only standard mental health care [36], and a psychological rehabilitation program versus conventional medical care [31]. A number of other studies (n = 3) compared different intervention between control and intervention groups: training for caregivers versus no intervention [37], time use intervention+ standard care versus self-activity education + standard care [32], and psychiatric telerehabilitation program versus conventional psychiatric rehabilitation [33]. In contrast, in the article that did not have a control group [34], all participants received the same intervention, which was pathways and resources for engagement and participation [34]. The occupational therapist led the intervention in some articles (n = 5) [32,33,34,35,37], but in others this was part of a multidisciplinary team (n = 2) [31,36] (Table 3).

The duration of the interventions ranged from less than 1 month (n = 4) [31,32,33,37] to 8 months [36]. In most articles, the total number of sessions was not stated (n = 3) [31,34,37]. In the rest, 7 sessions (n = 2) [32,33], 15 sessions (n = 1) [35], and 22 sessions (n = 1) [36] were carried out. Sessions lasted between 20 and 90 min (n = 4) [31,33,35,36], with the exception of one in which sessions lasted up to 3.5 h (n = 1) [37] and two in which the duration was not specified [32,34]. It should be noted that the lack of detailed information on session frequency and/or duration in some studies [31,32,34,37] limits comparability across interventions and should be considered when interpreting the results.

**Figure 3:**
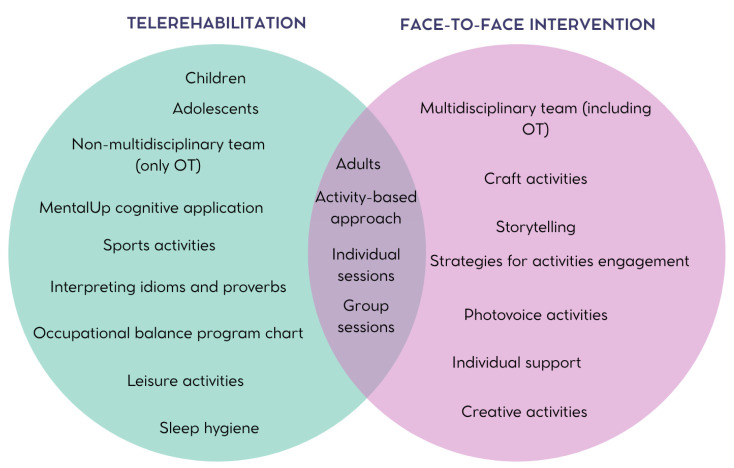
Comparison of OT interventions conducted via video call and in person.

The interventions were conducted via video call (n = 4) [33,34,35,37] or in person (n = 3) [31,32,36], including only individual sessions (n = 5) [31,32,33,34,37] or both individual and group sessions (n = 2) [35,36] (Table 3). Figure 3 presents a Venn diagram comparing OT interventions delivered via video call (telerehabilitation) and those delivered in person.

### 3.3. Study Variables and Measurement Instruments

Measurement tools used in the different studies are shown in Table 2. Quality of life [32,33,35,36], affective functions such as anxiety or depression [31,32,33,34], and sleep [31,32,33,37] were all evaluated in four articles. Participation in activities [34,36], boredom [32,33], and occupational balance [32,35] were assessed in two articles.

To assess participants’ quality of life, most studies used only one test, the Pediatric Quality of Life Inventory (PedsQL) [35] or The World Health Organization Quality of Life Assessment Instrument-BRIEF (WHOQOL-BREF) [32,33], while one study used two tools: the Manchester short assessment of quality of life (MANSA) [36] and EuroQol (EQ-5D-5L) for health-related quality of life [36]. To evaluate anxiety and depression, a different quantity of tools were used, ranging from a single tool, the BASC-3 test [34], to five tools [31]: two scales to assess anxiety, the Zung self-rating anxiety scale (SAS) and the visual analogue scale (VAS), and three to assess depression, the Zung self-rating depression scale (SDS), the patient health questionnaire-9 (PHQ-9), and the visual analogue scale (VAS). However, PHQ-9 [31,32,33] and SAS [31,32,33] were the most used tools to assess depression and anxiety, respectively. Sleep was evaluated for changes [37] and quality [31]. The sleep habits questionnaire (SHQ) and the consensus sleep diary (CSD) were used to observe changes in sleep patterns [37], while the Korean version of the insomnia severity index (ISI-K) was used to assess sleep quality [31,32,33].

Occupational balance was evaluated with the occupational balance questionnaire (OBQ11) [35] and the Korean Version of Life Balance Inventory (K-LBI) [32]. Participation in activities was measured using one evaluation tool per study: the Canadian occupational performance measure (COPM) [34], which measures both performance and satisfaction, and the profiles of occupational engagement in people with severe mental illness, self-reported version (POES-S) [36]. Finally, boredom was assessed in two articles using the multidimensional state boredom scale-8 (MSBS-8) [32,33].

Other study variables such as motor and cognitive functions [34], intrinsic motivation [35], general well-being [35], fear of COVID-19 [32], functioning (understood as cognition, mobility, self-care, interpersonal relationships, and daily activities) [36], and personal recovery [36] were evaluated in isolation in some studies using various questionnaires (Table 2).

### 3.4. Main Results of the Interventions

All the studies reported improved outcomes after the interventions in the experimental groups (n = 7) [31,32,33,34,35,36,37] (Table 3). In contrast, in two studies, the control group did not improve (n = 2) [35,37]. In one, participants in the control group were given online classes [35], while in the other, they received no intervention [37], which led to lower motivation, quality of life, functioning, well-being [35], and sleep [37]. However, the negative effect in the two studies was not statistically significant.

In general, the experimental group showed significantly better results than the control group (n = 3) [31,35,37]. However, in one study, there were no significant differences between the groups in terms of participation in activities, quality of life, functioning, or personal recovery [36], and interestingly, in another study, the intervention group showed higher anxiety and depression levels [33]. In this study, the control group received in-person psychiatric rehabilitation while the intervention group received telerehabilitation.

### 3.5. Main Limitations of the Included Studies

The most frequent limitation was the limited generalizability of the results (n = 5) [31,32,34,36,37]. Other frequently reported limitations were that self-reported measures were used (n = 3) [32,33,37], no random assignment of participants (n = 1) [34], short intervention periods (n = 1) [35], limited intervention activities (n = 1) [35], results were only measured twice (n = 1) [36], use of non-validated questionnaires (n = 1) [37], small sample size (n = 3) [32,33,37], and lack of participant follow-up (n = 2) [31,32]. None of the authors declared conflicts of interest (n = 7) [31,32,33,34,35,36,37], and five of the included studies received grants from different institutions [31,32,33,34,36] (Table 4).

## 4. Discussion

The interventions carried out during COVID-19 from OT in the field of mental health were OT via telerehabilitation, which was the most used; individual support; and education programs. In general, these interventions lasted between 1 to 32 weeks, involved approximately 14 sessions with durations ranging from 20 to 90 min, and were aimed at adults, adolescents, and children. The most addressed outcome variables were participation in activities, quality of life, anxiety, depression, and sleep problems.

In this scoping review, the included articles were published very recently, between 2022 and 2024, because COVID-19 emerged only four years ago. However, only five experimental studies [31,34,35,36,37] were found which investigated OT interventions in mental health during the lockdown. This scarcity may be due to a lockdown in many countries during the pandemic, which led to an unprecedented, rapid, and radical change in people’s lifestyles and rehabilitation. In addition, many occupational therapists lost their jobs during the pandemic [2]. All these implementation limitations may have contributed to the difficulty in conducting studies, not only in OT but in any health science discipline. In fact, most studies that we found which were conducted during the lockdown period were cross-sectional studies carried out through online surveys and questionnaires, retrospective studies, and pilot studies.

Occupational therapy via telerehabilitation was the most frequent intervention carried out during the COVID-19 lockdown in the field of OT and was primarily conducted with adults and children, and to a lesser extent with adolescents. As the lockdown prevented in-person interventions, the development of new treatment strategies such as telerehabilitation was necessary. The study by Ganesan et al. [9] showed that telerehabilitation was predominantly used in the treatment of children with Autism Spectrum Disorder. However, a review concluded that the use of this kind of rehabilitation was limited among individuals with mental health issues because of their problems using and accessing technologies [4,12]. Similarly, older adults or individuals with chronic illnesses, particularly those living in rural areas, did not benefit from telerehabilitation via videoconference because of its technological complexity and the high costs involved [38,39].

In this context, a recent review across health care professionals highlights that infrastructure limitations, technical challenges, psychological barriers, and workload-related concerns are common obstacles to digital interventions, whereas training, incentives, and perceived usefulness act as facilitators [15]. Additionally, a review in physiotherapy and OT indicate that telerehabilitation implementation raises equity and ethical challenges, including disparities in technology access, socioeconomic factors, geographic location, patient privacy, and autonomy [40]. More specifically, an integrative review on digital equity show that barriers disproportionately affect vulnerable groups, including low-income individuals, older adults, racial and ethnic minorities, immigrants and refugees, people with disabilities, and women, with major obstacles such as lack of internet access, digital literacy, language barriers, and internet costs [41]. Understanding the intersectionality of these factors is crucial for analyzing digital inequity and for designing telerehabilitation interventions that are accessible, fair, and effective for all populations.

A high number of included articles addressed participation in daily activities. The main reason for this could be that OT relies on the use of meaningful occupations and daily life activities to enhance health, increase participation, and improve treatment adherence [38,42]. OT constitutes a key intervention in the field of mental health and is typically used to improve social functioning, quality of life, and treatment adherence, as well as to reduce the number of readmissions among people with severe mental illness. In addition, the combined use of OT and telerehabilitation in mental health has been beneficial due to its feasibility, efficacy, and patient acceptance and satisfaction [12]. A review of articles focused on children and adolescents suffering from or at risk of developing mental health problems has shown that occupation-based interventions enhance social participation, behavior, and mental health [43].

In the interventions carried out in the included studies, the occupational therapist was in charge of analyzing daily activities to identify the skills required to perform them. The analysis involved assessing environmental barriers, creating routines, providing knowledge and strategies, and offering support to patients, which is in line with the role of occupational therapy [38]. However, during the pandemic, this role was compromised. A review of occupational therapists’ experiences during COVID-19 [2] revealed frustration due to a lack of resources, protocols, and evidence on how to conduct telerehabilitation, which restricted access and complicated the implementation of these strategies in clinical practice [2,38,39]. Another reason for this lack of implementation was that some occupational therapists had not received training in the use of telerehabilitation technologies [39].

The main objective of the included studies was to assess the impact of the interventions on participation in activities, quality of life, anxiety, depression, and sleep. This is in line with a previous review aimed at examining the effect of the COVID-19 lockdown on the mental health of children and adolescents, which found that anxiety and depression increased during the lockdown [21]. Similarly, a review on the impact of COVID-19 on individuals with eating disorders observed an increase in depression and anxiety during the pandemic, probably due to social isolation, changes in routine, and limited access to medical care [44]. The lockdown particularly triggered psychological distress among the most vulnerable groups, such as those with mental health issues [21]. These findings align with the scientific literature, which provides robust evidence showing that anxiety and depression were adversely affected during the pandemic, mainly as a result of isolation and lack of socialization [9,19,21,44,45,46,47,48].

Although the aim of this review is not to analyze the effectiveness of the interventions studied, we found it relevant to discuss the results reported by Jung and Ko, which suggested that psychological rehabilitation treatment for hospitalized patients had significant effects in both the psychiatric telerehabilitation (TR) and face-to-face conventional psychiatric rehabilitation (CR) groups, but the effect of TR was more limited than that of CR. Specifically, CR was more effective than TR in reducing anxiety and depression during hospitalization and had sustained effects at the 6-month follow-up. Several factors may explain this discrepancy. In CR, patients engaged in activities under the direct supervision of the occupational therapist, allowing nonverbal communication, real-time feedback, and the establishment of a stronger therapeutic alliance, which has been shown to enhance treatment outcomes [49,50]. In contrast, TR relied primarily on phone-based guidance, limiting visual cues such as eye contact, facial expressions, and gestures, which may have reduced patients’ sense of security and engagement [50,51], thereby reducing the effectiveness of the treatment [52]. Despite these limitations, TR still produced short-term improvements, likely due to the structured activity program and the formation of a basic therapeutic alliance [33,53]. Finally, although TR can present technical and security issues and limitations in therapeutic effects [54], it is a feasible and accessible intervention, particularly in contexts requiring social distancing, whose effectiveness may be enhanced by strategies that strengthen therapist–patient rapport and incorporate more interactive or visual communication elements.

This scoping review presents some limitations that could influence the results obtained. Firstly, selection bias is a major limitation which is common to all review studies. In this review, only articles published in English or Spanish were included, which may have increased selection bias and potentially led to the omission of relevant studies. This linguistic restriction could introduce geographical bias by excluding research from non-English/Spanish-speaking areas. However, English remains the predominant language in scientific publications, facilitating access to a large portion of the international literature. In this sense, it is worth noting that three of the seven included studies were conducted in Korea, showing that some evidence from non-English/Spanish contexts was still captured. The fact that nearly half of the included studies (3/7) originate from a single country may limit the external validity and generalizability of our conclusions. In addition, we only included articles where an occupational therapist was one of the professionals conducting the intervention, and thus possibly excluded articles where this was not specified, further contributing to selection bias. Secondly, not all articles measured the same variables or used the same evaluation tools. Although this review did not aim to analyze numerical results, the variety of measurement tools which were used complicated the comparison of results between studies and the extraction of conclusions. Consequently, the results of this review should be interpreted with some caution. Thirdly, the quality of the included articles was not assessed, as it is not a mandatory requirement for this type of review, which means some articles may have had low methodological quality. To minimize this limitation, a table summarizing the main limitations of the included articles, together with their funding sources and conflicts of interest, has been included to provide readers with information related to the quality of the studies. Fourthly, each country established different onset and duration dates to the lockdown periods, which made it complicated to establish a clear lockdown period common to all countries. To counteract this limitation, we included articles conducted during both the lockdown and the COVID-19 period in order to include the maximum number of articles possible. Fifthly, we cannot rule out publication bias, as no studies with null results have been included in this review.

Despite the above limitations, this scoping review has some strengths. Its primary strength is its novelty, as it studies the COVID-19 pandemic, which is a recent phenomenon. Moreover, no prior reviews describing OT interventions conducted during this period in mental health from OT were found. In addition, it is a specific review of OT, a relatively young discipline in terms of scientific research, and contributes to the development of the profession. This review addresses mental health, an especially relevant topic after the COVID-19 lockdown. In addition, the review includes experimental articles, a study design which provides the highest level of scientific evidence. Finally, this scoping review highlights several gaps in the current knowledge: (I) most included studies do not measure long-term intervention outcomes, which means more research and scientific evidence is needed; (II) no OT studies during COVID-19 addressing mental health in Spain were found; and (III) OT interventions are very scarce and vary significantly because they study different variables using a variety of questionnaires.

## 5. Conclusions

The interventions conducted in the field of mental health during COVID-19 by occupational therapists included OT via telerehabilitation as well as in person. These interventions lasted between 3 to 32 weeks; consisted of approximately 14 sessions ranging from 20 to 90 min; and were applied to adults, adolescents, and children. The most commonly addressed outcomes were participation in activities, quality of life, anxiety, depression, and sleep. The value of this scoping review lies in its rigorous methodology, its specific focus on OT, and the consolidation of evidence across diverse settings, which could serve as a guide for occupational therapists on how to implement interventions in the event of future pandemics or when in-person sessions are not possible due to accessibility issues or patient mobility limitations. Although the interventions could also be adapted for current practices, it is necessary to develop intervention protocols for clinical settings to promote evidence-based approaches in this field. It should be noted that we did not identify studies reporting null results, which suggests a possible publication bias. Therefore, our findings should be interpreted with caution and complemented with further evidence.

## Figures and Tables

**Table 1:** Databases and search strategies used.

Databases	Search Strategy 8 March 2025	Results
PubMed	“occupational therapy” [All Fields] AND “mental health” [All Fields] AND (“SARS CoV 2” [MeSH Terms] OR “SARS CoV 2” [All Fields] OR “COVID” [All Fields] OR “COVID 19” [MeSH Terms] OR “COVID 19” [All Fields])	276
Scopus	TITLE-ABS-KEY (“occupational therapy” AND “mental health” AND COVID)	129
Embase	(‘occupational therapy’/exp OR ‘occupational therapy’) AND (‘mental health’/exp OR ‘mental health’) AND (‘covid’/exp OR COVID)	375
Web of Science	“occupational therapy” AND “mental health” AND COVID (Topic)	118
OTSeeker	[Any Field] like ‘“occupational therapy”’ AND [Any Field] like ‘“mental health”’ AND [Any Field] like ‘COVID’	0
PsycINFO	“occupational therapy” AND “mental health” AND COVID	120

**Table 2:** Main characteristics of the studies included in this scoping review.

Author, Year	Design	Participants	Recruitment Period	Intervention/Comparator	Study Outcomes (Evaluation Tool)
Jung et al., 2022 [31]	RCT	109 isolated adult patients with COVID-19 (IG mean age: 51.06 years; CG mean age: 45.96 years)	From 27 May to 17 September 2021	Psychological rehabilitation program (n = 57)/conventional medical care (n = 52)	Anxiety (SAS, VAS). Depression (VAS, SDS, PHQ-9). Insomnia (ISI-K).
Belhan Çelik et al., 2022 [35]	RCT	52 refugee children aged between 13 and 15 years (IG mean age: 13.5 years; CG mean age: 13.5 years)	From 13 December to 31 December 2020	Online classes + OT via telerehabilitation (n = 26)/only online classes (n = 26)	Occupational Balance (OBQ11). Quality of life (PedsQL). Well-being (WSS). Intrinsic Motivation (IMS).
Bjørkedal et al., 2023 [36]	RCT	139 adults with psychiatric disabilities (IG mean age: 42 years; CG mean age: 44 years)	From September 2018 to August 2020	Meaningful activities and recovery (MA&R) + standard mental health care (n = 70)/only standard mental health care (n = 69)	Occupational engagement (POES-S). Functioning (WHODAS 2.0). Personal recovery (QPR). Quality of life (MANSA, EQ-5D-5L).
Jung et al. 2023 [32]	RCT	41 adult patients with COVID-19 (IG mean age: 47.37 years; CG mean age: 58.59 years)	From 1 February to 19 March 2021	Time use intervention + standard care (n = 19)/self-activity education + standard care (n = 22)	Occupational balance (K-LBI). Anxiety (SAS). Depression (PHQ-9). Boredom (MSBS-8). Insomnia (ISI-K). Quality of life (WHOQOL-BREF). Fear of COVID-19 (FCV-19S)
Anaby et al., 2024 [34]	nRCT	21 adolescents and young adults with physical disabilities (mean age: 21 years)	From August 2020 to November 2021	Pathways and resources for engagement and participation (PREP) (n = 21)/NA	Activity performance and satisfaction (COPM). Cognitive and affective functions (BASC-3). Motor functions (Trunk Impairment Scale, VAS, Goniometry, dynamometer, Finger dexterity test, Functional reach test, Berg Balance Scale and One-leg standing).
Leive et al., 2024 [37]	nRCT	30 children aged 3–10 years with neurodevelopmental disorder and insomnia (mean age: NS)	From June 2020 to September 2021	Program to support child sleep from the occupational therapy perspective [*Programa de Acompañamiento al Sueño en la Infancia desde Terapia Ocupacional*] (PASITO) (n = 22)/waiting list group (n = 8)	Sleep (SHQ, CSD).
Jung et al., 2024 [33]	RCT	40 adult patients with COVID-19 (IG mean age: 53.58 years; CG mean age: 49.06 years)	From 18 November 2021 to 7 April 2022	Psychiatric tele-rehabilitation program (n = 24)/conventional psychiatric rehabilitation (n = 16)	Anxiety (SAS, VAS). Depression (VAS, PHQ-9). Boredom (MSBS-8). Insomnia (ISI-K). Quality of life (WHOQOL-BREF).

BASC-3: behavior assessment system for children, third edition; COMP: Canadian occupational performance measure; CSD: consensus sleep diary; EQ-5D-5L: EuroQol-5 Dimensions-5 Levels; IMS: intrinsic motivation scale; FCV-19S: fear of COVID-19 scale; ISI-K: Korean version of the insomnia severity index; K-LBI: Korean Version of Life Balance Inventory; MANSA: Manchester short assessment of quality of life; MSBS-8: The multidimensional state boredom scale-8; nRCT: non-randomized controlled trial; OBQ11: occupational balance questionnaire; PedsQL: Pediatric Quality of Life Inventory; PHQ-9: patient health questionnaire-9; POES-S: profiles of occupational engagement in people with severe mental illness-self-rated version; QPR: questionnaire about processes of recovery; RCT: randomized controlled trial; SAS: Zung self-rating anxiety scale; SDS: Zung self-rating depression scale; SHQ: sleep habits questionnaire; VAS: visual analogue scale; WHODAS: World Health Organization Psychiatric Disability Assessment Schedule; WHOQOL-BREF: World Health Organization Quality of Life Assessment Instrument-BRIEF; WSS: Well-Star Scale.

**Table 3:** Summary of the interventions carried out in the studies included in this scoping review.

Author, Year	Intervention	Intervention Description	Intervention Duration	Professionals Involved in the Intervention	Main Results of the Interventions
Jung et al., 2022 [31]	In-person: psychological rehabilitation program in adults	An intervention consisting of education, craft activities, and physical activity. The education part included information about COVID-19. Craft activities included knitting, cross-stitching with jewelry, coloring, or block making. Physical activities included stretching, strength training, and breathing exercises.	Step 1: 20 min. Step 2: NS. Step 3: 8 days, 20 min per day (craft activities) and 8 days, 20 min per day (physical activity).	OT, nurse physician, psychiatrist, PT	IG and CG improved their levels of anxiety, depression, and insomnia after the interventions. Significantly decreased levels of anxiety, depression, and insomnia in IG vs. CG.
Belhan Çelik et al., 2022 [35]	Telerehabilitation: online classes + OT via telerehabilitation in children	Online classes: remote classes trough the Ministry of National Education Online Education Platform (EBA) as part of the children’s routine education plan. OT via telerehabilitation: it included both group and individual activities. Group activities were painting, sports, or games trough Zoom. Individual activities included routines and daily occupations organized in a balanced way; MentalUp application also used.	15 sessions, 3 weeks. Five 1 h session per week.	Investigator, OT	IG improved their occupational balance, well-being, intrinsic motivation, and health-related quality of life after the interventions. Only CG showed a significant decrease in the psychosocial health score after the intervention. Significantly improved occupational balance, well-being, intrinsic motivation, and health-related quality of life in IG vs. CG.
Bjørkedal et al., 2023 [36]	In-person: MA&R + standard health in adults	MA&R: Module I with two weekly sessions focusing on recognizing and exploring meaningful activities, and module II with two monthly sessions allowing participants to engage in new meaningful activities at their own pace. In addition, optional individual support to engage in activities was also offered. Standard mental health: The multidisciplinary Flexible Assertive Community Treatment model provided by Community Mental Health Services.	22 sessions, 8 months. Eleven group 90 min sessions and eleven individual 30–60 min sessions.	OT, peer worker	IG and CG improved their activity engagement, quality of life, and functioning after the interventions. They found no improvement in health-related quality of life in any of the groups. Only IG improved their personal recovery after the intervention. IG vs. CG: No significant differences were found between groups in activity participation, functioning, personal recovery, or quality of life after the interventions.
Jung et al., 2023 [32]	In-person: Time-use intervention + standard care	Patients and therapists analyzed daily activities in face-to-face sessions, selected meaningful occupations based on assessments, created timetables, and practiced selected tasks with therapist support and materials provided. Standard care included antiviral therapy for COVID-19.	A 7-day time-use intervention of four steps. Daily sessions.	OT	IG and CG reduced their depression and fear of coronavirus levels. Only CG showed significant reductions in boredom. IG vs. CG: IG had better results in occupational balance, insomnia, and quality of life.
Anaby et al., 2024 [34]	Telerehabilitation: PREP in adolescents and young adults	PREP is an individual-based intervention with five steps (make goals; map out a plan; make it happen; measure the process and outcomes; move forward), which focuses on modifying the environment. An OT met individually with each participant via video call to select a goal for participation in a leisure activity. The OT worked collaboratively with each participant to seek and create opportunities for participation in the chosen activity and to identify and remove potential environmental barriers for participation in that activity.	8-week self-chosen leisure activity at their home or community.	OT	An improvement in performance and satisfaction with chosen activities was observed after the intervention. A decrease in depression, anxiety, social stress, and hyperactivity was observed after the intervention. An improvement in at least one domain of body function occurred in 10 participants for motor outcomes.
Leive et al., 2024 [37]	Telerehabilitation: PASITO in children	The intervention objectives were based on the essential characteristics of sleep as an occupation promoting the implementation of adaptive and maladaptive strategies and sleep hygiene strategies adapted to the interests of each child and their primary caregivers. This intervention was remote, intensive, and individualized, coordinated by OTs and mediated by parents. The strategies were caregiver care, pleasant experiences, daily organization, routines and rituals, and rest.	Program of 19 days. Sessions of 3.5 h and/or 45 min.	OT, parents	IG improved their overall sleep, bedtime resistance, sleep onset, sleep duration, sleep anxiety, and night waking after the intervention. No significant changes in sleep were found in the CG after the intervention.
Jung et al., 2024 [33]	Telerehabilitation: psychiatric telerehabilitation program	Telephone-based telerehabilitation. Patients selected and performed daily 50 min meaningful activities (e.g., physical, craft, or leisure) with OT guidance. OTs provided remote support and monitored progress via daily phone calls, without direct contact.	Program of 7 days. Individual sessions of 50 min per day.	OT	IG and CG improved their quality of life and anxiety. Only CG showed significant reductions across all psychological distress measures. IG vs. CG: IG had higher levels of anxiety and depression.

CG: control group; IG: intervention group; MA&R: meaningful activities and recovery; NS: not stated; OT: occupational therapist; PASITO: program to support child sleep from the occupational therapy perspective; PREP: pathways and resources for engagement and participation, PT: physical therapist.

**Table 4:** Risk of bias of the included studies.

Author, Year	Main Limitations	Funding/Support	Conflicts of Interest
Jung et al., 2022 [31]	-Results may not reflect information from all patients, causing selection bias. -There were no baseline assessments of anxiety and depression levels. -Findings may be generalized to Korean patients, but they may be not applicable to other countries. -No follow-up of patients after discharge.	This research was supported by the Accountable Care Hospital Connected Care (ACHCC) Project funded by the Ministry of Health and Welfare of Korea (Project Number: 2022-ACHCC-L26).	None declared.
Belhan Çelik et al., 2022 [35]	-The intervention period was relatively short. -Authors did not assess the impact of their favorable results on learning ability or school success. -The activities used in the intervention were limited.	The authors received no financial support.	None declared.
Bjørkedal et al., 2023 [36]	-No blinding of participants or staff to intervention allocation. -Outcome measures consisted of self-report instruments that are more prone to bias. -Outcomes were only measured at baseline and after the intervention. -Low generalizability of the results. The trial was partially conducted during the COVID-19 pandemic, and results may be unique to this setting. -No specific data were obtained on participants’ use of services relative to standard mental health care, nor on the type of care participants received.	The Tryg Foundation (ID number 112526), the Research Foundation of the Danish Occupational Therapy Association (FF 117 R45 A1271), and a grant from the Mental Health Services of the Capital Region of Denmark sponsored the RCT.	None declared.
Jung et al., 2023 [32]	-Small sample size. -Study conducted at a single center; therefore, the results cannot be generalized. -Self-reported questionnaires were used as outcome measurements. -The possibility of socially desirable responses, recall bias, and misunderstanding of questions cannot be excluded. -Follow-up after discharge was not performed. -The non-blinded trial design could have caused performance bias and detection bias.	This research was supported by the Ministry of Education of the Republic of Korea and the National Research Foundation of Korea (NRF-2021S1A3A2A02096338)	None declared.
Anaby et al., 2024 [34]	-Low generalizability of the results. Only participants with physical disabilities and without intellectual delay were included. -Participants were not randomly assigned. -Exploratory analysis of the impact of functional limitations and mental health problems were not adjusted for multiple comparisons.	Institute of Human Development, Child and Youth Health of the Canadian Institutes of Health Research. Grant Number: 166213	None declared.
Leive et al., 2024 [37]	-Objective measures were not used. -The sleep habits questionnaire has not been validated in the Argentine population. -The small sample size, which does not guarantee generalization of the results. -The time availability of the caregivers and internet connection may imply a bias in terms of families having a high-income level. -The small size of the control group made comparison with the intervention group difficult.	The authors received no financial support.	None declared.
Jung et al., 2024 [33]	-Small sample size. -Patients’ baseline mental health scores were unknown. -The PHQ-9 score in the intervention group at admission was significantly higher than the corresponding score in the control group. -Self-reported evaluation scale was used in this study. -Recall bias, misinterpretations, and socially desirable responses cannot be ruled out.	This research was supported by the Accountable Care Hospital Connected Care (ACHCC) Project funded by the Ministry of Health and Welfare of Korea (Project Number:2024-ACHCC-L26).	None declared.

## Data Availability

The original contributions presented in this study are included in the article/Supplementary Materials. Further inquiries can be directed to the corresponding author(s).

## Supplementary Materials

The following supporting information can be downloaded at: https://www.mdpi.com/article/10.3390/healthcare13172136/s1, Table S1: PRISMA-ScR checklist.

## Abbreviations

The following abbreviations are used in this manuscript:

COVID-19	Coronavirus disease
PRISMA-ScR	PRISMA extension for Scoping Reviews
OT	Occupational Therapy
WHO	World Health Organization

## References

[1] Onyeaka H., Anumudu C.K., Al-Sharify Z.T., Egele-Godswill E., Mbaegbu P. (2021). COVID-19 Pandemic: A Review of the Global Lockdown and Its Far-Reaching Effects. Sci. Prog..

[2] Ranjan G.K., Gandhi S., Sivakumar T. (2024). Experiences of the Occupational Therapists During the COVID-19 Pandemic: A Scoping Review. J. Psychosoc. Rehabil. Ment. Health.

[3] Moss B.P., Mahajan K.R., Bermel R.A., Hellisz K., Hua L.H., Hudec T., Husak S., McGinley M.P., Ontaneda D., Wang Z. (2020). Multiple Sclerosis Management during the COVID-19 Pandemic. Mult. Scler..

[4] Muralidar S., Ambi S.V., Sekaran S., Krishnan U.M. (2020). The Emergence of COVID-19 as a Global Pandemic: Understanding the Epidemiology, Immune Response and Potential Therapeutic Targets of SARS-CoV-2. Biochimie.

[5] Ochani R., Asad A., Yasmin F., Shaikh S., Khalid H., Batra S., Sohail M.R., Mahmood S.F., Ochani R., Hussham Arshad M. (2021). COVID-19 Pandemic: From Origins to Outcomes. A Comprehensive Review of Viral Pathogenesis, Clinical Manifestations, Diagnostic Evaluation, and Management. Infez. Med..

[6] Khunti K., Valabhji J., Misra S. (2023). Diabetes and the COVID-19 Pandemic. Diabetologia.

[7] Reyes-Sánchez F., Basto-Abreu A., Torres-Alvarez R., Canto-Osorio F., González-Morales R., Dyer-Leal D.D., López-Ridaura R., Zaragoza-Jiménez C.A., Rivera J.A., Barrientos-Gutiérrez T. (2022). Fraction of COVID-19 Hospitalizations and Deaths Attributable to Chronic Diseases. Prev. Med..

[8] Chapman K.M., Berger M.J., Doherty C., Anastakis D.J., Baltzer H.L., Boyd K.U., Bristol S.G., Byers B., Chan K.M., Cunningham C.J.B. (2021). Recommendations for Patients with Complex Nerve Injuries during the COVID-19 Pandemic. Can. J. Neurol. Sci..

[9] Ganesan B., Fong K.N.K., Meena S.K., Prasad P., Tong R.K.Y. (2021). Impact of COVID-19 Pandemic Lockdown on Occupational Therapy Practice and Use of Telerehabilitation—A Cross Sectional Study. Eur. Rev. Med. Pharmacol. Sci..

[10] Robinson M.R., Koverman B., Becker C., Ciancio K.E., Fisher G., Saake S. (2021). Lessons Learned From the COVID-19 Pandemic: Occupational Therapy on the Front Line. Am. J. Occup. Ther..

[11] Tenforde A.S., Borgstrom H., Polich G., Steere H., Davis I.S., Cotton K., O’Donnell M., Silver J.K. (2020). Outpatient Physical, Occupational, and Speech Therapy Synchronous Telemedicine: A Survey Study of Patient Satisfaction with Virtual Visits During the COVID-19 Pandemic. Am. J. Phys. Med. Rehabil..

[12] Sánchez-Guarnido A.J., Domínguez-Macías E., Garrido-Cervera J.A., González-Casares R., Marí-Boned S., Represa-Martínez Á., Herruzo C. (2021). Occupational Therapy in Mental Health via Telehealth during the COVID-19 Pandemic. Int. J. Environ. Res. Public. Health.

[13] von Zweck C., Naidoo D., Govender P., Ledgerd R. (2023). Current Practice in Occupational Therapy for COVID-19 and Post-COVID-19 Conditions. Occup. Ther. Int..

[14] Hung Kn G., Fong K.N. (2019). Effects of Telerehabilitation in Occupational Therapy Practice: A Systematic Review. Hong Kong J. Occup. Ther..

[15] Borges do Nascimento I.J., Abdulazeem H., Vasanthan L.T., Martinez E.Z., Zucoloto M.L., Østengaard L., Azzopardi-Muscat N., Zapata T., Novillo-Ortiz D. (2023). Barriers and Facilitators to Utilizing Digital Health Technologies by Healthcare Professionals. npj Digit. Med..

[16] Lee V., Albaum C., Tablon Modica P., Ahmad F., Gorter J.W., Khanlou N., McMorris C., Lai J., Harrison C., Hedley T. (2021). The Impact of COVID-19 on the Mental Health and Wellbeing of Caregivers of Autistic Children and Youth: A Scoping Review. Autism Res..

[17] Jin Y., Murray L. (2023). Perinatal Mental Health and Women’s Lived Experience of the COVID-19 Pandemic: A Scoping Review of the Qualitative Literature 2020–2021. Midwifery.

[18] Ren X., Huang W., Pan H., Huang T., Wang X., Ma Y. (2020). Mental Health During the Covid-19 Outbreak in China: A Meta-Analysis. Psychiatr. Q..

[19] Sideli L., Lo Coco G., Bonfanti R.C., Borsarini B., Fortunato L., Sechi C., Micali N. (2021). Effects of COVID-19 Lockdown on Eating Disorders and Obesity: A Systematic Review and Meta-Analysis. Eur. Eat. Disord. Rev..

[20] Richter D., Riedel-Heller S., Zürcher S.J. (2021). Mental Health Problems in the General Population during and after the First Lockdown Phase Due to the SARS-Cov-2 Pandemic: Rapid Review of Multi-Wave Studies. Epidemiol. Psychiatr. Sci..

[21] Panchal U., Salazar de Pablo G., Franco M., Moreno C., Parellada M., Arango C., Fusar-Poli P. (2023). The Impact of COVID-19 Lockdown on Child and Adolescent Mental Health: Systematic Review. Eur. Child. Adolesc. Psychiatry.

[22] Sessford J.D., Dodwell A., Elms K., Gill M., Premnazeer M., Scali O., Roque M., Cameron J.I. (2025). Factors Associated with Mental Health Outcomes among Family Caregivers to Adults with COVID: A Scoping Review. Disabil. Rehabil..

[23] Safieh J., Broughan J., McCombe G., McCarthy N., Frawley T., Guerandel A., Lambert J.S., Cullen W. (2022). Interventions to Optimise Mental Health Outcomes During the COVID-19 Pandemic: A Scoping Review. Int. J. Ment. Health Addict..

[24] Higgins J.P.T., Thomas J., Chandler J., Cumpston M., Li T., Page M.J., Welch V.A. (2023). Cochrane Handbook for Systematic Reviews of Interventions Version 6.4.

[25] Tricco A.C., Lillie E., Zarin W., O’Brien K.K., Colquhoun H., Levac D., Moher D., Peters M.D.J., Horsley T., Weeks L. (2018). PRISMA Extension for Scoping Reviews (PRISMA-ScR): Checklist and Explanation. Ann. Intern. Med..

[26] Bramer W.M., Rethlefsen M.L., Kleijnen J., Franco O.H. (2017). Optimal Database Combinations for Literature Searches in Systematic Reviews: A Prospective Exploratory Study. Syst. Rev..

[27] Pollock D., Peters M.D.J., Khalil H., McInerney P., Alexander L., Tricco A.C., Evans C., de Moraes É.B., Godfrey C.M., Pieper D. (2023). Recommendations for the Extraction, Analysis, and Presentation of Results in Scoping Reviews. JBI Evid. Synth..

[28] Arksey H., O’Malley L. (2005). Scoping Studies: Towards a Methodological Framework. Int. J. Soc. Res. Methodol..

[29] Peters M.D.J., Marnie C., Tricco A.C., Pollock D., Munn Z., Alexander L., McInerney P., Godfrey C.M., Khalil H. (2020). Updated Methodological Guidance for the Conduct of Scoping Reviews. JBI Evid. Synth..

[30] Levac D., Colquhoun H., O’Brien K.K. (2010). Scoping Studies: Advancing the Methodology. Implement. Sci..

[31] Jung J.H., Won J.J., Ko J.Y. (2022). Psychological Rehabilitation for Isolated Patients with COVID-19 Infection: A Randomized Controlled Study. PLoS ONE.

[32] Jung J.H., Ko J.Y., Hong I., Jung M.-Y., Park J.-H. (2023). Effects of a Time-Use Intervention in Isolated Patients with Coronavirus Disease 2019: A Randomized Controlled Study. PLoS ONE.

[33] Jung J.H., Ko J.Y. (2024). Depression, Anxiety and Insomnia among Isolated Covid-19 Patients: Tele Occupational Therapy Intervention vs. Conventional One: A Comparative Study. BMC Psychol..

[34] Anaby D.R., Avery L., Palisano R.J., Levin M.F., Khayargoli P., Hsieh Y.-H., Gorter J.W., Teplicky R. (2024). BEYOND Consultant Team Environment-Based Approaches to Improve Participation of Young People with Physical Disabilities during COVID-19. Dev. Med. Child. Neurol..

[35] Belhan Çelik S., Özkan E., Bumin G. (2022). Effects of Occupational Therapy via Telerehabilitation on Occupational Balance, Well-Being, Intrinsic Motivation and Quality of Life in Syrian Refugee Children in COVID-19 Lockdown: A Randomized Controlled Trial. Children.

[36] Bjørkedal S.-T.B., Bejerholm U., Hjorthøj C., Møller T., Eplov L.F. (2023). Meaningful Activities and Recovery (MA&R): A Co-Led Peer Occupational Therapy Intervention for People with Psychiatric Disabilities. Results from a Randomized Controlled Trial. BMC Psychiatry.

[37] Leive L., Melfi D., Lipovetzky J., Cukier S., Abelenda J., Morrison R. (2024). Program to Support Child Sleep from the Occupational Therapy Perspective during the COVID-19 Pandemic. Arch. Argent. Pediatr..

[38] Gately M.E., Metcalf E.E., Waller D.E., McLaren J.E., Chamberlin E.S., Hawley C.E., Venegas M., Dryden E.M., O’Connor M.K., Moo L.R. (2023). Caregiver Support Role in Occupational Therapy Video Telehealth: A Scoping Review. Top. Geriatr. Rehabil..

[39] Abbott-Gaffney C.R., Gafni-Lachter L., Cason J., Sheaffer K., Harasink R., Donehower K., Jacobs K. (2022). Toward Successful Future Use of Telehealth in Occupational Therapy Practice: What the COVID-19 Rapid Shift Revealed. Work.

[40] Veras M., Sigouin J., Auger L.-P., Auger C., Ahmed S., Boychuck Z., Cavallo S., Lévesque M., Lovo S., Miller W.C. (2025). A Rapid Review of Ethical and Equity Dimensions in Telerehabilitation for Physiotherapy and Occupational Therapy. Int. J. Environ. Res. Public. Health.

[41] Raihan M.M.H., Subroto S., Chowdhury N., Koch K., Ruttan E., Turin T.C. (2025). Dimensions and Barriers for Digital (in)Equity and Digital Divide: A Systematic Integrative Review. DTS..

[42] Dehghani S., Mirzakhany N., Dehghani S., Pashmdarfard M. (2023). The Use of Tele-Occupational Therapy for Children and Adolescents with Different Disabilities: Systematic Review of RCT Articles. Med. J. Islam. Repub. Iran..

[43] Cahill S.M., Egan B.E., Seber J. (2020). Activity- and Occupation-Based Interventions to Support Mental Health, Positive Behavior, and Social Participation for Children and Youth: A Systematic Review. Am. J. Occup. Ther..

[44] Devoe D.J., Han A., Anderson A., Katzman D.K., Patten S.B., Soumbasis A., Flanagan J., Paslakis G., Vyver E., Marcoux G. (2023). The Impact of the COVID-19 Pandemic on Eating Disorders: A Systematic Review. Int. J. Eat. Disord..

[45] Grumi S., Provenzi L., Gardani A., Aramini V., Dargenio E., Naboni C., Vacchini V., Borgatti R. (2021). Engaging with Families through On-line Rehabilitation for Children during the Emergency (EnFORCE) Group Rehabilitation Services Lockdown during the COVID-19 Emergency: The Mental Health Response of Caregivers of Children with Neurodevelopmental Disabilities. Disabil. Rehabil..

[46] Dhiman S., Sahu P.K., Reed W.R., Ganesh G.S., Goyal R.K., Jain S. (2020). Impact of COVID-19 Outbreak on Mental Health and Perceived Strain among Caregivers Tending Children with Special Needs. Res. Dev. Disabil..

[47] Farajzadeh A., Dehghanizadeh M., Maroufizadeh S., Amini M., Shamili A. (2021). Predictors of Mental Health among Parents of Children with Cerebral Palsy during the COVID-19 Pandemic in Iran: A Web-Based Cross-Sectional Study. Res. Dev. Disabil..

[48] Nisticò V., Bertelli S., Tedesco R., Anselmetti S., Priori A., Gambini O., Demartini B. (2021). The Psychological Impact of COVID-19-Related Lockdown Measures among a Sample of Italian Patients with Eating Disorders: A Preliminary Longitudinal Study. Eat. Weight. Disord..

[49] Flückiger C., Del Re A.C., Wampold B.E., Horvath A.O. (2018). The Alliance in Adult Psychotherapy: A Meta-Analytic Synthesis. Psychotherapy.

[50] Lin T., Stone S.J., Heckman T.G., Anderson T. (2021). Zoom-in to Zone-out: Therapists Report Less Therapeutic Skill in Telepsychology versus Face-to-Face Therapy during the COVID-19 Pandemic. Psychotherapy.

[51] Burgoyne N., Cohn A.S. (2020). Lessons from the Transition to Relational Teletherapy During COVID-19. Fam. Process.

[52] Henry S.G., Fuhrel-Forbis A., Rogers M.A.M., Eggly S. (2012). Association between Nonverbal Communication during Clinical Interactions and Outcomes: A Systematic Review and Meta-Analysis. Patient Educ. Couns..

[53] Aafjes-Van Doorn K., Békés V., Luo X., Hopwood C.J. (2024). Therapists’ Perception of the Working Alliance, Real Relationship and Therapeutic Presence in in-Person Therapy versus Tele-Therapy. Psychother. Res..

[54] Kafali N., Cook B., Canino G., Alegria M. (2014). Cost-Effectiveness of a Randomized Trial to Treat Depression among Latinos. J. Ment. Health Policy Econ..

